# The Diminution of R‐Loops Generated by LncRNA DSP‐AS1 Inhibits DSP Gene Transcription to Impede the Re‐Epithelialization During Diabetic Wound Healing

**DOI:** 10.1002/advs.202406021

**Published:** 2025-02-07

**Authors:** Chen Yang, Hong Lian, Hengli Luo, Chenlin Song, Jianghong Lin, Zhuoxian Liang, Yulin Yang, Xiaosi Hong, Shaohua Li, Yanbo Chen, Liangyan Wu, Li Yan, Sifan Chen, Meng Ren

**Affiliations:** ^1^ Department of Endocrinology Sun Yat‐sen Memorial Hospital Sun Yat‐sen University 107 Yanjiang West Road Guangzhou 510120 China; ^2^ Guang Dong Clinical Research Center for Metabolic Diseases Guangzhou 510120 China; ^3^ Department of Endocrinology and Metabolism Zhuhai People's Hospital (The Affiliated Hospital of Beijing Institute of Technology, Zhuhai Clinical Medical College of Jinan University) Zhuhai 519000 China; ^4^ Department of Chemical and Systems Biology Stanford University Stanford CA 94305 USA; ^5^ Wards of Cadres Zhuhai People's Hospital (The Affiliated Hospital of Beijing Institute of Technology, Zhuhai Clinical Medical College of Jinan University) Zhuhai 519000 China; ^6^ Guangdong Provincial Key Laboratory of Malignant Tumor Epigenetics and Gene Regulation Guangdong‐Hong Kong Joint Laboratory for RNA Medicine Medical Research Center Sun Yat‐sen Memorial Hospital Sun Yat‐sen University Guangzhou 510120 China; ^7^ Nanhai Translational Innovation Center of Precision Immunology Sun Yat‐sen Memorial Hospital Foshan 528200 China

**Keywords:** anti‐sense lncRNAs, desmoplakin, diabetic wound healing, R‐loop, ten‐eleven translocation dioxygenase proteins

## Abstract

Re‐epithelialization constitutes a critical stage in the intricate process of wound healing, yet its mechanisms in the context of diabetic wounds remain elusive. In this study, the role of the mesenchymal‐epithelial transition (MET) vis‐à‐vis the epithelial‐mesenchymal transition (EMT) of keratinocytes in diabetic wound re‐epithelialization is investigated. The findings reveal an impediment in the MET process, rather than EMT, which significantly compromised re‐epithelialization in diabetic wounds. Furthermore, Desmoplakin (*DSP*) gene expression, encoding a key desmosome protein, is down‐regulated in diabetic rats. This down‐regulation coincided with aberrant hypo‐demethylation of the *DSP* promoter. The inhibition of *DSP* expression is linked to reduced occupancy of Ten‐eleven translocation 3 (TET3) at the *DSP* promoter, consequently suppressing TET3‐dependent DNA demethylation. Additionally, a novel lncRNA termed DSP‐AS1is identified, which is antisense to *DSP*. Notably, DSP‐AS1 expression is down‐regulated in diabetic skin wounds, and it interacted with TET3, a DNA demethylase. Notably, DSP‐AS1 is found to form R‐loops, triple‐stranded DNA:RNA hybrids, at the *DSP* promoter, facilitating TET3 localization to the *DSP* promoter. Collectively, the findings suggest that reduced R‐loop formation by DSP‐AS1 impairs *DSP* gene transcription by repressing TET3‐mediated DNA demethylation. This disruption of the orchestrated re‐epithelialization process contributes to refractory diabetic wound healing.

## Graphical abstract

Down‐regulated LncRNA DSP‐AS1, the diminution of R‐loop formation, reduced Ten‐eleven translocation 3 (TET3) occupancy, inhibited DNA demethylation at the DSP promoter, and disrupted re‐epithelialization process proceed sequentially during delayed diabetic wound healing. R‐loops serve as a reliable epigenetic hallmark for TET localization to mediate demethylation.

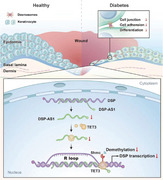

## 1 Introduction

Diabetic foot ulcers (DFUs), characterized by delayed wound healing, are a major complication of diabetes mellitus (DM).^[^ ^1^ ^]^ Cutaneous wound healing is a well‐orchestrated process involving distinct yet overlapping phases: hemostasis, inflammation, proliferation, re‐epithelialization, and remodeling.^[^ ^2^ ^]^ Re‐epithelialization, the dynamic process of resurfacing a wound with new epithelium, is critical for wound healing.^[^ ^3^ ^]^ Nevertheless, the status of re‐epithelialization during diabetic wound healing remains enigmatic.

In the conventional course of wound healing, epidermal keratinocytes at the wound periphery undergo epithelial‐to‐mesenchymal transition (EMT), characterized by the loss the epithelial markers (including E‐cadherin (E‐cad), desmoplakin (DSP), par‐3 family cell polarity regulator (PARD3), etc.) and the acquisition of mesenchymal traits (including N‐cadherin (N‐cad), vimentin (VIM), fibronectin 1 (FN1), etc.). This transition enables keratinocytes to dismantle cell‐cell junctions acquire a fibroblastic morphology, and migrate to close the wound.^[^ ^4^, ^5^ ^]^ Subsequently, these cells must undergo a reversal of this process, termed mesenchymal‐to‐epithelial transition (MET), to reestablish an epithelial phenotype and generate secondary epithelial layers.^[^ ^6^, ^7^ ^]^ In this study, we reveal impediments in the re‐epithelialization process during the late stages of diabetic wound healing, implicating the down‐regulation of DSPs as a potential causative factor. DSP serves as a crucial linker protein between the intermediate filament scaffolding and the desmosomal plaque, essential for stabilizing the cytoskeleton^[^ ^8^ ^]^ and maintaining normal desmosomal adhesion.^[^ ^9^ ^]^ Our research delves into the underlying mechanisms responsible for DSP down‐regulation.

In mammals, active DNA demethylation mediated by Ten‐eleven translocation dioxygenase proteins (TETs) represents a prominent of gene transcription regulation.^[^ ^10^ ^]^ TET activity is linked to various aspects of DM, including susceptibility, insulin resistance, and diabetic complications such as DFUs.^[^ ^11^ ^]^ Long non‐coding RNAs (lncRNAs), transcripts exceeding 200 bases in length, are a significant class of non‐protein coding RNAs. Functioning as epigenetic regulators, lncRNAs modulate transcriptional and post‐transcriptional control processes by interacting with proteins and nucleic acids involved in gene expression regulation, thereby participating in the pathogenesis of numerous human diseases.^[^ ^12^ ^]^ Growing evidence suggests the potential role of lncRNAs as key regulators in diabetic wound healing.^[^ ^13^, ^14^, ^15^ ^]^ Notably, anti‐sense lncRNAs inherently possess sequence elements that can base‐pair with their cognate RNA, thus exhibiting the capability to regulate their anti‐sense genes at the transcription level in *cis*.^[^ ^16^ ^]^ Nonetheless, their precise function in diabetic wound healing remains poorly understood.

## 2 Results

### 2.1 Down‐Regulation of *DSP* During Cutaneous Wound Healing in Diabetic Rats

**Figure 1:**
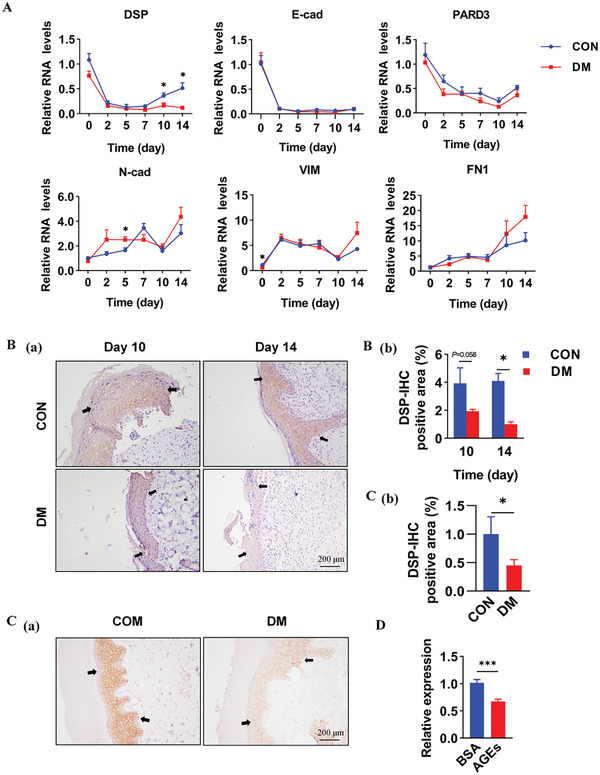
DSP downregulation during the late stage of diabetic wound healing. A): RT‐qPCR analysis depicting the mRNA expression levels of desmoplakin (*Dsp*), E‐cadherin (*E‐cad*), Par‐3 family cell polarity regular (*Pard3*), N‐cadherin (*N‐cad*), vimentin (*Vim*), and febronectin1 (*Fn1*) in skin wound tissues of control and diabetic rats on days 0, 2, 5, 7, 10, and 14 of wound healing (*n* = 4). β‐actin (ACTB) served as an internal control. B,C): Immunohistochemical (IHC) staining of DSP in skin tissues of (B) diabetic rats, (C) diabetic patients, and their respective controls. Quantitative analysis of DSP protein levels (brown color) in the epidermis was conducted based on integrated optical density (IOD) (*n* = 4). Arrows indicate the edge of epithelialization. Scale bar, 200 µm. D): RT‐qPCR analysis of *Dsp* mRNA expression levels in the human keratinocyte cell lines (HaCaT) cells treated with either BSA solution (100 mm) or AGEs‐BSA (100 mm) for 72 h (*n* = 3). ACTB served as an internal control. Data are presented as mean ± SD, and *P* values were calculated using a two‐sided *t*‐test. ^*^ *P* < 0.05, ^***^ *P* < 0.001.

To assess wound healing dynamics, skin wound models were established in non‐diabetic (control, CON) and diabetic (DM) rats. As expected, diabetic wounds exhibited delayed healing compared to controls (Figure S1A,B, Supporting Information). Tissue samples were collected from rats at various time points from Day 0 to Day 14, and the expression levels of genes indicative of epithelialization status were analyzed, including epithelial markers (*Dsp*, *E‐cad*, *Pard3*) and mesenchymal markers (*N‐cad*, *Vim*, *Fn1*) (**Figure** **1**A). Additionally, the expression of other EMT‐related genes, such as claudin (*Cldn1*), keratin 5 (*Krt5*), keratin 13 (*Krt13*), snail family transcriptional repressor 2 (*Snai2*), and dystonin (*Dst*), was examined (Figure S1D, Supporting Information).

In both non‐diabetic and DM rats, the expression levels of *N‐cad* and *FN1* generally exhibited an increasing trend during wound healing. Meanwhile, *VIM* expression showed a transient rise at the beginning of wound healing (Day 0), followed by a decline on Day 2–10, and another increase by Day 14. Conversely, the mRNA levels of all epithelial markers in control rats declined sharply on Day 2 and remained low until Day 7–10 before recovering (Figure 1A). While the trends for epithelial markers were similar in diabetic wounds, *Dsp* expression exhibited a robust rebound at the late stage (Day 14) in control rats, which was absent in diabetic wounds, where *Dsp* remained significantly lower (2–5 folds, *p <* 0.05, Figure 1A). These findings suggest aberrant down‐regulation of *Dsp* specifically during the late stage of wound healing in diabetic rats, potentially contributing to impaired re‐epithelialization.

Further examination of DSP protein expression in skin tissues of diabetic rats via immunohistochemistry (IHC) revealed the primary localization of DSP in the epidermis, and its expression level at the late stage of wound healing was considerably lower in DM rats compared to CON (*P* < 0.05, Figure 1B). Similar results were obtained with human skin wound tissues; DSP expression was significantly lower in DM patients compared to controls (*P* < 0.05, Figure 1C). In vitro experiments using human keratinocyte cell lines (HaCaT) mimicked diabetic conditions with advanced glycation end products (AGEs) administration and showed a subsequent down‐regulation of DSP mRNA levels (*P* < 0.001, Figure 1D). Additionally, no significant changes in *Dsp* expression were observed in other organs, such as heart, liver, and kidney, between diabetic and non‐diabetic rats (Figure S1E(a), Supporting Information).

### 2.2 The Down‐Regulation of DSP Impairs Cutaneous Wound Healing

**Figure 2:**
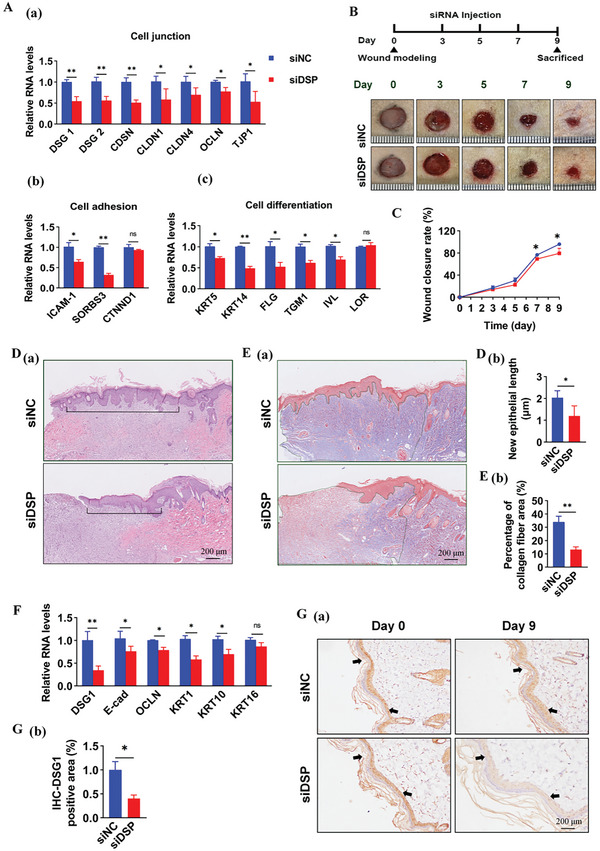
DSP down‐regulation impairs normal wound healing. A): Effects of DSP silencing on the mRNA expression of cell junction‐related genes, including desmoglein 1 (*Dsg1*), desmoglein 2 (*Dsg2*), corneodesmosin (*Cdsn*), claudin 1 (*Cldn1*), claudin 1 (*Cldn4*), occludin (*Ocln*), tight junction protein 1 (*Tjp1*), cell adhesion‐related genes, including intercellular adhesion molecule 1(*Icam‐1*), sorbin, SH3 domain containing 3 (*Sorbs3*), and catenin delta 1 (*Ctnnd1*), and cell differentiation‐related genes, including keratin 5 (*Krt5*), keratin 14 (*Krt14*), filaggrin (*Flg*), transglutaminase 1(*Tgm1*), involucrin (*Ivl*), and loricrin (*Lor*) in HaCaT cells. B) (upper panel): Schematic representation of the experimental workflow involving siRNA injection after wound creation in normal rats. B (lower panels): Representative images of cutaneous wounds in normal rats treated with control siRNAs (siNC) and siRNAs target to DSP (siDSP) on days 0, 3, 5, 7, and 9 post‐wounding (middle panel). C) Wound closure rates were quantified using ImageJ Pro Plus software and expressed as the percentage of closed wound area (*n* = 5) (lower panel). D,E): Representative images of skin tissues from normal rats treated with siNC or siDSP for nine days, stained with (D) H&E and (E) Masson's trichome to visualize the epidermis and collagen content in the dermis, respectively. Re‐epithelization length and the percentage of collagen fiber area were quantified using ImageJ Pro Plus software (*n* = 5). F): RT‐qPCR analysis of three cell junction‐related genes (*Dsg1*, *E‐cad*, *Ocln*) and three cell differentiation‐related genes (*Krt1*, *Krt10*, *Krt16*) in skin tissues of normal rats treated with siNC and siDSP, respectively (*n* = 5). G): IHC staining of DSG1 protein (brown color) in the epidermis and quantitative analysis of DSG1 expression in skin tissues of normal rats treated with siNC or siDSP (*n* = 5) using ImageJ Pro Plus software. Data are presented as mean ± SD. *P* values were calculated using a two‐sided *t*‐test. ^*^ *P* < 0.05, ^**^ *P* < 0.01, NS (not significant, *P* > 0.05).

To elucidate the role of DSP in regulating cell connection, adhesion, and differentiation— crucial processes for re‐epithelialization—we silenced DSP in HaCaT cells using siRNAs targeting *DSP* (siDSP). Knockdown of DSP by siDSP was verified (*P <* 0.001, Figure S2A,B, Supporting Information). DSP silencing led to down‐regulation of genes related to cell connection (desmoglein 1 (*DSG1*), desmoglein 2 (*DSG2*), corneodesmosin (*CDSN*), *CLDN1*, *CLDN4*, occludin (*OCLN*), and tight junction protein 1 (*TJP1*)), adhesion (intercellular adhesion molecule 1(*ICAM1*), sorbin, SH3 domain containing 3 (*SORBS3*) and catenin delta 1 (*CTNND1*)), and differentiation (*KRT5*, *KRT14*, filaggrin (*FLG)*, transglutaminase 1(*TGM1*), involucrin (*IVL*), and loricrin (*LOR*)), suggesting that down‐regulated DSP may inhibit these processes (**Figure** **2**A). However, DSP silencing did not affect cell apoptosis (Figure S2C, Supporting Information), cell cycle (Figure S2F, Supporting Information), or cell migration (Figure S2E, Supporting Information), while there was only a modest alteration in cell proliferation (Figure S2D, Supporting Information).

To assess the impact of *Dsp* down‐regulation on skin wound healing, a local knockdown of *Dsp* was performed using siDSP around skin wounds in non‐diabetic rats (Figure 2B). qPCR and IHC assays confirmed successful down‐regulation of *Dsp* by siDSP (Figure S2G, I, Supporting Information). Wound healing was observed to be significantly delayed in the siDSP group compared to controls (Figure 2B,C). Notably, *Dsp* silencing did not induce histological changes in other organs such as the heart, liver, spleen, lung, and kidney by H&E staining (Figure S2H, Supporting Information), nor did it significantly alter plasma biochemical indexes (Table S3, Supporting Information). Furthermore, H&E and Masson staining revealed a considerable reduction in secondary epithelial length and collagen deposition in the dermis upon *Dsp* silencing (Figure 2D,E), suggesting impaired re‐establishment of the regenerative epidermis and collagen deposition. Consistent with in vitro data, silencing DSP down‐regulated genes related to cell junction (*Dsg1* and *Ocln*), adhesion (*E‐cad*), and differentiation (*Krt1*, *Krt10*, and *Krt16*) (Figure 2F). IHC staining further confirmed a significant decrease in the expression of DSG1, a component of intercellular desmosome junctions, upon DSP silencing (Figure 2G).

Furthermore, the CRISPR/Cas9‐based Synergistic Activation Mediator (SAM) system by GeneChem Technology (Shanghai, China) was employed to up‐regulate endogenous DSP in HaCaT cells (dCAS9‐VP64‐Puro virus)^[^ ^17^ ^]^ and overexpression of DSP was verified (*P* < 0.001, Figure S3A, B, Supporting Information). Overexpression of DSP via the SAM system resulted in the up‐regulation of genes associated with cell junction, adhesion, and differentiation (Figure S3C, Supporting Information), without cell apoptosis, cell cycle, proliferation, or migration (Figure S3 D–G, Supporting Information). Additionally, analysis of gene expression and protein levels via RT‐qPCR and IHC in diabetic rat skin revealed significant down‐regulation of cell junction, adhesion, and differentiation‐related genes (Figure S1 F,G, Supporting Information), such as DST, CLDN1, KRT5, KRT13, and KRT17, except for E‐cad which showed a slight non‐significant increase (Figure S1G, Supporting Information). IHC staining supported these findings, demonstrating down‐regulation of DSG1, OCLN, CTNND1, and KRT10, with no significant changes in the expression of E‐cad, KRT1, ICAM‐1, and KRT16 in DM rats compared to controls (Figure S1F, Supporting Information). Collectively, these data underscore the critical role of DSP down‐regulation in delayed cutaneous wound healing.

### 2.3 TET3‐Mediated Demethylation of the DSP Promoter

**Figure 3:**
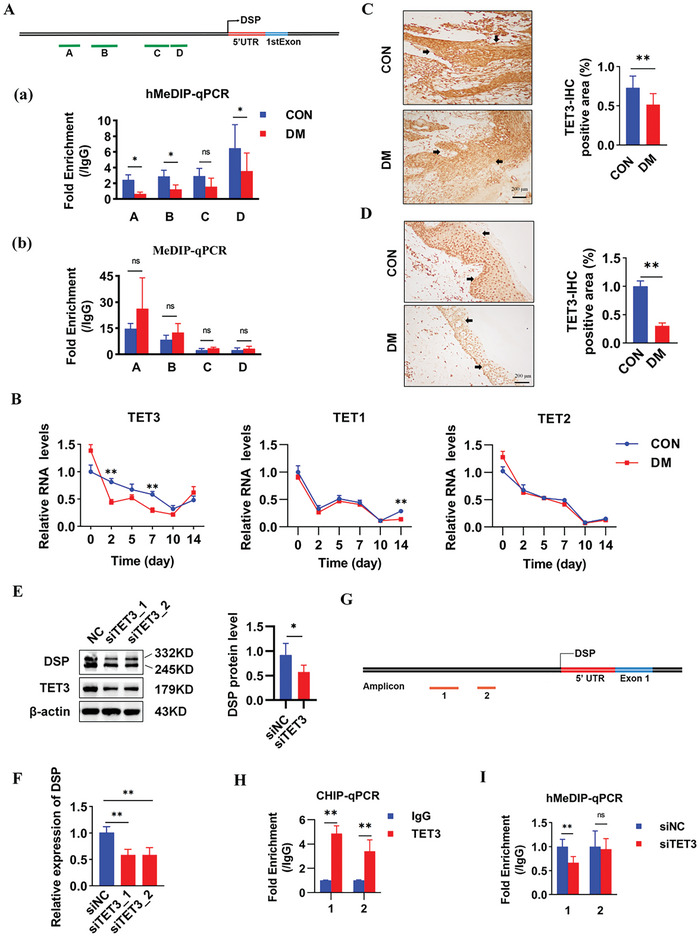
TET3 promotes DSP expression by mediating demethylation at the DSP promoter. A) (upper panel): Schematic representation of the four DSP amplicons targeting the DSP promoter region in rats. (a,b): hMeDIP (a) and subsequent MeDIP‐qPCR (b) analysis to assess the demethylation and methylation levels at the DSP promoter region in skin wound tissues of control and diabetic rats on day 14 of wound healing (*n* = 4). B RT‐qPCR analysis of TET3, TET2, and TET1 expression in skin wound tissues of control and diabetic rats during wound healing (*n* = 4). C,D): IHC staining of TET3 in skin wound tissues of (C) diabetic rats and (D) diabetic patients, and their respective controls. Quantitative analysis was performed using ImageJ Pro Plus software (*n* = 4). E,F): Effects of TET3 silencing on DSP expression levels at the (E) protein and (F) mRNA levels, evaluated by in vitro RT‐qPCR and Western blot, respectively. G): Schematic representation of the 2 amplicons within the DSP gene used in Figure H&I. Sites 1 and 2 were located in the DSP promoter region. H): In vitro ChIP‐qPCR analysis monitoring TET3 occupancy at the two amplicons of *Dsp* gene, respectively. I Effects of TET3 silencing on the demethylation level at the two amplicons of *Dsp* gene, respectively, assessed by in vitro hMeDIP‐qPCR analysis. Data are presented as mean ± SD. *P* values were calculated using a two‐sided *t*‐test. ^*^ *P* < 0.05, ^**^ *P* < 0.01, NS (not significant, *P* > 0.05).

Metabolic disorders like hyperglycemia can alter DNA demethylation rates and influence gene expression.^[^ ^18^ ^]^ Examination of the *DSP* gene revealed the presence of CpG islands at its promoter, suggesting a propensity for DNA demethylation^[^ ^18^ ^]^ (Figure S4A, Supporting Information). To investigate the methylation and demethylation status at the *Dsp* promoter in diabetic rats, hydroxymethylated DNA immunoprecipitation‐quantitative polymerase chain reaction (hMeDIP‐qPCR) and methylated DNA immunoprecipitation‐qPCR (MeDIP‐qPCR) were employed. Six primer pairs were designed for qPCR after methylated immunoprecipitation (IP), covering ≈90% of the DSP promoter region (Table S5, Supporting Information). After excluding areas lacking significant enrichment, four selected amplicons exhibited notable enrichment of 5‐hydroxymethylcytosine (5hmC) and 5‐methylcytosine (5mC) enrichment compared with IgG (Figure S4B,C, Supporting Information). The results revealed a significant decrease in 5hmC levels at sites A (−1315–−1502 bp) and B (−981–−1211 bp) of the *Dsp* promoter (*P <* 0.05, **Figure** **3**A), accompanied by elevated 5mC levels at site B (*P <* 0.05, Figure 3A (a,b)). TET enzymes function as “methylation erasers”, catalyzing the oxidation of 5mC to 5hmC, while DNA methyltransferases (DNMTs) act as “methylation writers” and maintainers, transferring methyl groups derived from S‐adenosylmethionine (SAM).^[^ ^18^, ^19^ ^]^ The mRNA expression levels of three predominant TETs (TET1, TET2, and TET3) (Figure 3B) and three predominant DNMTs (DNMT1, DNMT3A, and DNMT3B) were evaluated (Figure S4D, Supporting Information). TET1 and TET3 were found to be down‐regulated during diabetic wound healing. Given the negligible expression of TET1 in skin tissue (https://www.proteinatlas.org/search/TET1), focus was then placed on investigating the potential regulatory role of TET2 and TET3 in *DSP* expression. While knockdown of TET2 had no impact on DSP expression (data not shown), silencing TET3 in vitro significantly down‐regulated both DSP mRNA and protein levels (Figure 3E,F; Figure S4E, Supporting Information), suggesting a crucial role for TET3 in passive demethylation at the *DSP* promoter. IHC results confirmed the down‐regulation of TET3 protein in skin tissues of diabetic rats (Figure 3C) and diabetic patients (Figure 3D).

Subsequently, chromatin immunoprecipitation (ChIP)‐qPCR assays confirmed TET3 occupancy specifically at site 1 (−1247 – −1061 bp) and site 2 (−1561–−1463 bp) within the *DSP* promoter (Figure 3H). Silencing of TET3 resulted in reduced 5hmC levels at the *DSP* promoter at site 1 (Figure 3I), accompanied by shortened G0/G1 cell cycle phase, prolonged S phase, and inhibition of both cell proliferation and migration (Figure S4F–H, Supporting Information). In summary, our findings suggest that TET3‐mediated demethylation is essential for DSP expression, highlighting the critical role of TET3 in this regulatory process.

### 2.4 lnRNA DSP‐AS1 Mediates TET3 Recruitment and Demethylation of the DSP Promoter

**Figure 4:**
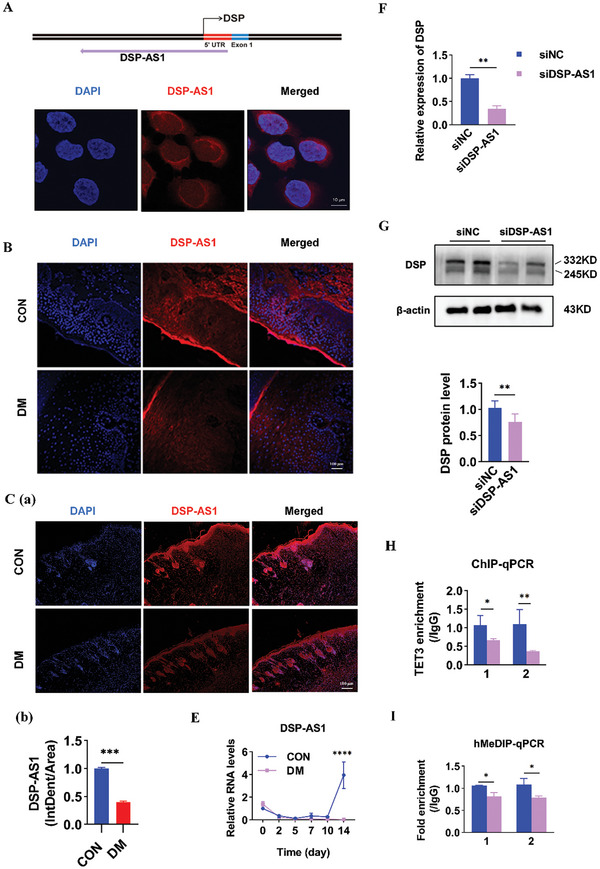
DSP‐AS1 recruits TET3 to mediate demethylation at the DSP promoter. A) (upper panel): Schematic diagram illustrating the region on the *DSP* gene where DSP‐AS1 is transcribed from the antisense strand. A (lower panel): Representative image of FISH analysis for cellular localization of DSP‐AS1 (red) in HaCaT cells. Nuclei were stained with DAPI (blue). Scale bar, 10 µm. B): FISH analysis of DSP‐AS1 expression in skin wound tissues of non‐diabetic (*n* = 7) and diabetic patients (*n* = 6), respectively. Scale bar, 100 µm. C): FISH analysis of DSP‐AS1 expression in skin wound tissues of control and diabetic rats on day 14 of wound healing (*n* = 4). Scale bar, 100 µm. E): RT‐qPCR analysis of DSP‐AS1 expression levels in skin wound tissues of control and diabetic rats on days 0, 2, 5, 7, 10, and 14 of wound healing (*n* = 4). F): RT‐qPCR analysis of DSP expression following DSP‐AS1 silencing in HaCaT cells. G): Western blot analysis of DSP expression following DSP‐AS1 silencing in HaCaT cells. H): ChIP‐qPCR analysis to evaluate the enrichment level of TET3 at sites 1 and 2 of DSP promoter after DSP‐AS1 silencing in HaCaT cells. I): hMeDIP‐qPCR analysis to evaluate the demethylation level at sites 1 and 2 of DSP promoter after DSP‐AS1 silencing in HaCaT cells. Data are present as mean ± SD. *P* values were calculated using a two‐sided t‐test. ^*^ *P* < 0.05, ^**^ *P* < 0.01, ^***^ *P* < 0.001.

Following the validation of TET3 recruitment to the *DSP* promoter for demethylation, we aimed to elucidate the mechanism underlying the specific localization of TET3 to this promoter region. Recent studies have underscored the role of lncRNAs as scaffolds in recruiting regulatory complexes, including TET enzymes, to specific genomic loci through the formation of DNA: lncRNA: protein complexes.^[^ ^20^, ^21^ ^]^ Through online databases, a novel and suitable lncRNA antisense to *DSP*, named DSP‐AS1, with the potential to regulate *DSP* gene transcription, was identified (**Figure** **4**A). To validate the expression of DSP‐AS1 in HaCaT cells, RNA fluorescence in situ hybridization (RNA‐FISH) was performed, revealing the expression of DSP‐AS1 in both the nucleus and cytoplasm (Figure 4A). Additionally, RT‐qPCR assays demonstrated significantly lower levels of DSP‐AS1 in skin wound tissues of diabetic rats on day 14 of wound healing compared to controls (Figure 4E). Further RNA FISH assays detected lower levels of DSP‐AS1 in diabetic tissues from both DM patients and rats compared to controls (Figure 4B&C), indicating down‐regulation of DSP‐AS1 in diabetic wounds.

To determine the function of DSP‐AS1, DSP‐AS1 was silenced in vitro. This resulted in down‐regulation of DSP expression (Figure 4F&G), a significant reduction in TET3 enrichment at the *DSP* promoter (Figure 4H), and decreased 5hmc enrichment at both sites 1 and 2 (Figure 4I). Taken together, our data suggest that DSP‐AS1‐dependent recruitment of TET3 is essential for demethylation of the *DSP* promoter.

### 2.5 DSP‐AS1 Recruits TET3 via R‐Loop Formation

**Figure 5:**
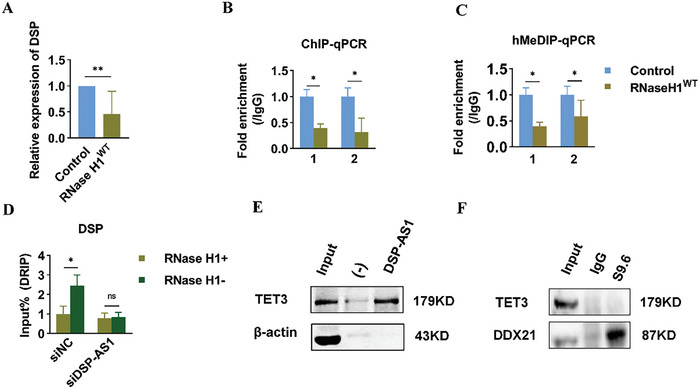
R‐loops generated by DSP‐AS1 mediate the recruitment of TET3 to the *DSP* promoter. A): RT‐qPCR analysis of DSP mRNA expression levels following overexpression of RNAaseH1^WT^ in HaCaT cells. B): ChIP‐qPCR analysis to evaluate the enrichment level of TET3 at sites 1 and 2 of *DSP* promoter after overexpressing RNAaseH1^WT^ in HaCaT cells. C): hMeDIP‐qPCR analysis to evaluate the demethylation level at sites 1 and 2 of the DSP promoter after RNAaseH1^WT^ overexpression in HaCaT cells. D): DRIP‐qPCR analysis to monitor R‐loops at the *DSP* promoter in HaCaT cells treated with siNC and siDSP‐AS, with or without RNAaseH1 enzyme treatment, respectively. “Rnase H1+” refers to the addition of exogenous RNase H1 enzymes, while “Rnase H1‐“ indicates no addition of exogenous RNase H1 enzymes. E): Coimmunoprecipitation (Co‐IP) experiment demonstrating the interaction of TET3 or DDX21 with endogenous R‐loops. Cellular R‐loops were immunoprecipitated by the S9.6 antibody, and co‐precipitated DDX21 and TET3 were detected by Western blotting. “(‐)” refers to a random single‐stranded sequence that acts as a negative control probe for DSP‐AS1. F): RNA pull‐down assays to assess the occupancy of DSP‐AS1 at TET3. Data are presented as mean ± SD (*n* = 3 biological replicates). *P* values were calculated using a two‐sided t‐test. ^*^ *P* < 0.05, ^**^ *P* < 0.01, NS (not significant, *P* > 0.05).

Having identified DSP‐AS1 as a mediator of TET3 recruitment and *DSP* promoter demethylation, our next step was to investigate the mechanism underlying targeted recruitment of *DSP* promoter by the DSP‐AS1‐TET3 complex. DSP‐AS1 is transcribed in the antisense direction to *DSP*, overlapping with a CpG island (CGI) at the *DSP* promoter (https://genome.ucsc.edu). Analysis of the *DSP* gene locus revealed a GC skew partially overlapping with DSP‐AS1 (http://rloop.bii.a‐star.edu.sg), potentially favoring R‐loop formation.^[^ ^22^ ^]^ R‐loops are three‐stranded structures formed by DNA:RNA hybrids and a displaced single‐stranded DNA (ssDNA) molecule. They frequently arise during transcription and can influence gene expression by interfering with DNA replication, repair, and transcription.^[^ ^23^ ^]^ Recently, R‐loops have been implicated in regulating gene expression by limiting DNA methylation.^[^ ^24^, ^25^ ^]^ To assess R‐loop formation at the *DSP* promoter, DNA:RNA immunoprecipitation (DRIP) assays using the antibody S9.6, which recognizes R‐loops, were performed. The results confirmed the presence of R‐loop structures at the locus where TET3 was enriched (**Figure** **5**A). Overexpression of wild‐type RNAse H1 (RNAaseH1^WT^), an enzyme that degrades R‐loops, led to a reduction in *DSP* mRNA levels (Figure 5A). Moreover, overexpression of RNAaseH1^WT^ significantly decreased TET3 enrichment at site 1 (Figure 5B). Subsequently, hMeRIP‐qPCR analysis of the *DSP* promoter revealed that overexpressing RNAaseH1^WT^ reduced the enrichment of 5hmc at site 1 (Figure 5C). After silencing DSP‐AS1, DRIP‐qPCR assays showed a reduction in R‐loop levels at the *DSP* promoter (Figure 5D).

To investigate whether TET‐3 is directly bound to the R‐loop structure, Coimmunoprecipitation (Co‐IP) assays were performed using DDX21 as a positive control, which efficiently binds to R‐loops.^[^ ^26^ ^]^ However, TET3 failed to interact with the R‐loop‐antibody S9.6 complex, suggesting that TET3 does not bind to R‐loop structures directly (Figure 5F). Consistent with this finding, RNA pull‐down assays indicated that TET3 is bound to DSP‐AS1 (Figure 5E). Collectively, these results suggest that DSP‐AS1 facilitates TET3 recruitment to the *DSP* promoter by forming R‐loops, thereby promoting demethylation.

## 3 Discussion

### 3.1 Graphical Overview

Down‐regulated LncRNA DSP‐AS1, the diminution of R‐loop formation, reduced Ten‐eleven translocation 3 (TET3) occupancy, inhibited DNA demethylation at the DSP promoter, and disrupted re‐epithelialization process proceeded sequentially during delayed diabetic wound healing. R‐loops serve as a reliable epigenetic hallmark for TET localization to mediate demethylation.

The re‐epithelialization status during diabetic wound healing remains elusive. In this study, we demonstrate that the re‐epithelialization process in diabetic wound healing is hindered. Importantly, we identify the retardation of MET as a novel contributing factor to abnormal diabetic wound healing. Intriguingly, as a critical component of desmosomes, DSP is found to be aberrantly down‐regulated. The depletion of DSP results in impaired restoration of epithelial integrity and disrupts the coordinated re‐epithelialization process. Furthermore, the attenuated recruitment of TET3 leads to hypo‐demethylation at the *DSP* promoter, thereby inhibiting *DSP* expression. This down‐regulation of *DSP* is originally induced by the down‐regulation of DSP‐AS1, an lncRNA that interacts with TET3. R‐loops generated by DSP‐AS1 serve as a reliable hallmark for the localization of TET3 at the *DSP* promoter to mediate demethylation. Our findings offer new insights into the pathogenesis of chronic diabetic wounds.

The dynamics of re‐epithelialization during diabetic wound healing remain unexplored in the literature. Herein, we characterized that the re‐epithelialization process was disorganized during diabetic wound healing. Especially, concomitant with the upregulation of epithelial markers, a conspicuous upward trend in mesenchymal markers was observed from Day 10 of wound healing. One possible explanation was that the wounds were still quite large on day 10–14 of wound healing in diabetic rats (Figure S1B, Supporting Information), which required a higher level of mesenchymal markers to maintain the migration of epithelial cells to close the wound.^[^ ^12^ ^]^ Additionally, FN1 could promote the proliferation of fibroblasts and facilitate the contraction,^[^ ^10^ ^]^ contributing to wound healing at a late stage, which might lead to an increase in FN1 expression. A previous study suggested that promoting EMT of keratinocytes could enhance diabetic wound healing.^[^ ^27^ ^]^ Therefore, it was previously assumed that EMT was inhibited during diabetic wound healing. Surprisingly, our data uncover that it is MET, rather than EMT, that is impeded. Under favorable conditions, MET enables keratinocytes to generate secondary epithelia and restore barrier function. Here, we identify the retardation of MET as a critical new contributor to abnormal diabetic wound healing. Moreover, the essential desmosome component DSP is found to be aberrantly down‐regulated. Dysfunction of DSP has been associated with skin lesions and loss of hair integrity.^[^ ^28^, ^29^, ^30^ ^]^ Additionally, DSP plays a crucial role in maintaining airway epithelial integrity, and its low expression level is implicated in the development of idiopathic pulmonary fibrosis.^[^ ^31^ ^]^ Similarly, DSP deficiency in esophageal epithelial cells may exert a detrimental effect on epithelial homeostasis, contributing to the pathogenesis of eosinophilic esophagitis.^[^ ^32^ ^]^ Moreover, DSP mutations cause a unique form of arrhythmogenic cardiomyopathy characterized by a high prevalence of left ventricular fibrosis and systolic dysfunction.^[^ ^32^ ^]^

Impaired DSP may lead to epidermal instability, resulting in suprabasal clefting and acantholysis throughout the spinous layer in vivo, highlighting the crucial role of DSP in maintaining epidermal integrity.^[^ ^29^, ^33^ ^]^ Our data demonstrate that the down‐regulation of DSP inevitably compromises the restoration of epithelial integrity and disrupts the coordinated re‐epithelialization process by interfering with epidermal cell‐cell junctions and adhesion, thus contributing to chronic and refractory wound healing. Paradoxically, DSP silencing slightly inhibited both cell proliferation and adhesion, while did not exert an impact on cell migration (Figure S2E, Supporting Information). It was reported that the inhibition of cell adhesion would facilitate cell migration,^[^ ^12^ ^]^ whereas the inhibition of cell proliferation could hinder cell migration.^[^ ^9^ ^]^ Hence, we propose that when silencing DSP, due to the inhibition of both cell adhesion and cell proliferation, cell migration did not exhibit significant changes.

Metabolic perturbations, including hyperglycemia, have been shown to affect the rate of DNA demethylation and alter gene transcription activity by regulating the activity of enzymes involved in the balance between DNA methylation and demethylation.^[^ ^18^ ^]^ We observed that the *DSP* promoter was hypomethylated under diabetic conditions, and the repression of TET3‐dependent demethylation inhibited the expression of *DSP*, suggesting a critical role for TET3‐mediated demethylation in regulating *DSP* expression during wound healing.

TET proteins function as “methylation erasers”, which utilize the tricarboxylic acid (TCA) cycle intermediate α‐ketoglutarate to remove methyl groups.^[^ ^18^ ^]^ TET3‐mediated DNA repair mechanism can further achieve the demethylation process by removing 5hmC and further oxidation products through thymine DNA glycosylase (TDG) combined with base excision repair (BER) or nucleotide excision repair (NER) mechanism.^[^ ^34^, ^35^ ^]^ TET proteins require localization to specific genomic regions for targeted demethylation.^[^ ^10^ ^]^ TET proteins are recruited by transcription factors and by RNA polymerase II to modify 5mC at enhancers and gene bodies, thereby regulating gene expression.^[^ ^36^ ^]^ Our study reveals a novel recruitment mechanism whereby DSP‐AS1 facilitates TET3 binding to the *DSP* promoter, thereby promoting demethylation. Given that DSP‐AS1 remained at a low level during the late stages of diabetic wound healing, we hypothesized that decreased DSP‐AS1 contributed to the down‐regulation of DSP through attenuated recruitment of TET3 to the *DSP* promoter. However, the key aspect of this regulatory mechanism lies in how the lncRNA and the demethylation machinery are localized to the specific locus. In the present study, we observed a GC skew within the *DSP* promoter that overlaps with DSP‐AS1, potentially faciliating R‐loop formation.^[^ ^37^ ^]^ Indeed, R‐loop structures were present at the *DSP* promoter. It has been reported that R‐loop formation promotes transcription activity by protecting CpG islands (CGIs) from DNA methylation at unmethylated human promoters.^[^ ^38^ ^]^ Additionally, DNA methyltransferases have poor affinity for RNA/DNA hybrids, allowing R‐loops to hinder methylation‐mediated transcriptional silencing.^[^ ^39^ ^]^ However, the role of R‐loops in regulating DNA methylation/demethylation remains largely unclear. Our results further provide evidence that R‐loops generated by DSP‐AS1 are essential for TET3‐specific recruitment to the *DSP* promoter, although TET3 does not directly bind to the R‐loop structure. Paradoxically, Arab et al. reported that GADD45A (Growth Arrest and DNA Damage Protein 45A), a demethylation machinery, directly binds to generic DNA:RNA hybrids and mediates local DNA demethylation by recruiting TET1.^[^ ^22^ ^]^ These contrasting observations highlight the intricate nature of R‐loop interactions with TET proteins. Collectively, our study unveils the critical role of R‐loops in regulating DNA demethylation, with R‐loops serving as a reliable epigenetic hallmark for TET localization to mediate demethylation, despite the demethylation machinery not directly binding to the R‐loop structure.

In summary, our study highlights that the down‐regulation of DSP is implicated in impaired re‐epithelialization and delayed diabetic wound healing. We suggest that the decrease in DSP‐AS1, an epigenetic regulator, results in diminished recruitment of TET3 to the *DSP* promoter, leading to the subsequent down‐regulation of DSP. R‐loops generated by DSP‐AS1 play a crucial role in the specific recruitment of TET3 to the *DSP* promoter. These findings hold significant implications for understanding the pathogenesis and potential therapeutic approaches for chronic diabetic wounds.

## 4 Experimental Section

### Materials

The human keratinocyte cell lines (HaCaT) were obtained from Procell Life Science & Technology (CL‐0090). Small interfering RNAs (siRNAs) designed to target DSP mRNA, TET3 mRNA, and cytoplasmic DSP‐AS1, as well as antisense oligonucleotides (ASO) targeting nuclear DSP‐AS1, along with their corresponding negative controls (NC), were synthesized by RiboBio (Guangzhou, China). Additionally, plasmids encoding RNasseH1^WT^ and RNasseH1^D209N^ were generously provided by Professor Hongjie Yao of Guangzhou Institute of Biomedicine and Health, Chinese Academy of Sciences (GIBH), Guangzhou, China. The antibodies and critical experimental reagents utilized in our study are listed in Table S1 (Supporting Information).

### Cell Culture and Treatment

The HaCaT cells were maintained in Minimum Essential Medium (MEM; Procell Life Science & Technology, US) supplemented with 10% fetal bovine serum (Gibco, US) and 50 U mL^−1^ of penicillin/streptomycin (Gibco, US). Cells in log‐phase were harvested using trypsin‐EDTA solution (Gibco, US). Primary rat epidermal keratinocytes were isolated from the skin tissue of newborn Sprague‐Dawley rats with Dispase II (Roche, US), and cultured with keratinocyte‐defined medium (KM; ScienCell, US) supplemented with Keratinocyte Growth Supplement‐defined (ScienCell, US) and penicillin/streptomycin solution (ScienCell, US). All cells were incubated under standard culture conditions (5% CO_2_ and 95% humidified air at 37 °C). HaCaT cells were treated with 100 µg mL^−1^ advanced glycation end products (AGEs)‐BSA or BSA (Merck Millipore, Temecula, CA) for 72 h. To manipulate gene expression, HaCaT cells were transfected with siRNAs targeting DSP or TET3 mRNA, or NC siRNA using Lipofectamine 3000 Transfection kit (Thermofisher, US) according to the manufacturer's instructions. The CRISPR/Cas9‐based Synergistic Activation Mediator (SAM) system from GeneChem Technology (Shanghai, China) was employed to up‐regulate endogenous DSP in HaCaT cells. Briefly, dCAS9‐VP64‐Puro lentivirus was used to infect HaCaT cells.^[^ ^17^ ^]^ After infection for 48 h, 2 g mL^−1^ puromycin (T19978, TargetMol) was added. Subsequently, cells were cultured with a half‐dose of puromycin and infected with sgRNA‐MS2‐P65‐HSF1 lentivirus (Shanghai Genechem Co., Ltd) for 48 h. After treatment with 20µg mL^−1^ G418 for 48 h, DSP over‐expressing cells were selected. Western blot analysis confirmed successful DSP overexpression, and these cells were used for downstream experiments.

### RNA Isolation and Quantitative Real‐Time PCR Analysis

Total RNA was extracted from cells using TRIzol reagent (Takara Bio Inc. Tokyo, Japan) following the manufacturer's instructions. Subsequently, reverse transcription was performed using the *Evo* M‐MLV RT Premix kit, and RT‐qPCR was performed using the SYBR Green Premix Pro Taq HS qPCR Kit (Accurate Biotechnology, Hunan, China) according to the manufacturer's instructions. Relative RNA expression levels were normalized to the housekeeping gene β‐actin (ACTB) for quantification. The primers used in real‐time PCR are listed in Table S2a (Supporting Information).

### Protein Extraction and Western Blot Analysis

Total protein was extracted from HaCaT cells and skin tissues using RIPA lysis buffer (CWBiotech, Beijing, China) supplemented with a protease and phosphatase inhibitor cocktail (CWBiotech, Beijing, China). Protein concentration was determined using the BCA protein assay kit (CWBiotech, Beijing, China). Equal amounts of total protein (20 µg) were separated by SDS‐PAGE on 7.5% gels (EpiZyme, Shanghai) and transferred to polyvinylidene fluoride (PVDF) membranes (Merck Millipore, USA). After blocking with 5% non‐fat milk for 1 h, the membranes were incubated with primary antibodies overnight at 4 °C. Following washes with PBST, the membranes were incubated with secondary antibodies for 1 h at room temperature. Protein bands were visualized using the Mini Chemi imaging system (Sage Creation) and quantified with Image‐Pro Plus Software 6.0 (Media Cybernetics, CA, US). Protein levels were normalized to ACTB for quantification.

### Immunohistochemistry (IHC)

The skin tissues were fixed overnight at 4 °C in 4% paraformaldehyde before paraffin embedding. Sections of 4 µm thickness were collected for further analysis. IHC assays were performed as previously described. Briefly, the slides were deparaffinized and dewaxed to water through a series of washes, then baked at 65 °C for 2 h. Antigen retrieval was achieved by boiling the slides in pH 9.0 EDTA solution (Servicebio, Wuhan, China) for 15 min at sub‐boiling temperature. After cooling to room temperature, endogenous peroxidase activity was quenched with 3% H_2_O_2_ for 10 min at room temperature (Beyotime Biotechnology, China). The slides were then incubated with primary antibodies at 4 °C overnight. The following day, slides were incubated with a secondary antibody at 37 °C for 1 h, and the color was developed using DAB (CWBiotech, Beijing, China) working solution. Images were captured using an Olympus IX71 microscope. Five fields from each section were observed and analyzed.

### Hematoxylin and Eosin (H&E) and Masson's Trichrome Staining

Skin and visceral tissues were fixed overnight at 4 °C in 4% paraformaldehyde and then embedded in paraffin. Skin sections were stained with both H&E and Masson's trichrome staining (Solarbio, Beijing, China) according to the manufacturer's instructions. For H&E staining, the edge of scars and wound areas were measured using ImageJ software. The percentage of re‐epithelialization was calculated using the equation: E% = WN/WO × 100, where WO represents the original wound area and WN denotes the length of newly generated epithelium across the wound surface. Masson's trichrome staining was conducted to analyze the degree of collagen maturity in the wound beds.

### Cell Proliferation Assay

Cell proliferation was assessed using a Cell Counting Kit‐8 (CCK‐8) assay (CWBiotech, Beijing, China) following the manufacturer's instructions. HaCat cells were transfected with NC and siDSP, respectively. At 0, 24, 48, 72, and 96 h post‐treatment, optical density (OD) was quantified at 450 nm using a microplate reader (TECAN Spark10 m).

### Wound‐Healing Assay in Vitro

HaCaT cells were seeded in 6‐well plates and transfected with NC and siDSP, respectively, for 48 h. Then, the cells were scratched with a 200‐µL pipette tip along the ruler. Samples were observed and photographed under white light with a microscope (Olympus IX71) at 0 and 24 h. The wound widths were measured with Image‐Pro Plus Software 6.0 (Media Cybernetics, CA, US), and the percentage of wound healing was calculated using the following formula: (0 h scratch wounding width‐24 h scratch wounding width)/ (0 h scratch wounding width) × 100%.^[^ ^40^ ^]^

### Flow Cytometric Analysis of Apoptosis and Cell Cycle

After 48 h of treatment with NC and siDSP, respectively, HaCat cells were harvested for flow cytometry analyses of apoptosis and cell cycle alterations. Apoptosis was quantified using the Annexin V‐FITC/Propidium Iodide (AV/PI) Apoptosis Detection Kit (CWBiotech, Beijing, China) according to the manufacturer's protocols. Cell cycle analysis was performed using a cell cycle analysis PI kit (CWBiotech, Beijing, China). Apoptosis and cell cycle alteration data were analyzed using flow cytometry (Becton Dickinson, Franklin Lakes, NJ, USA).

### RNA Fluorescence In Situ Hybridization (FISH)

The FISH assay was performed to determine the cellular localization and the expression of DSP‐AS1 using the Fluorescence in Situ Hybridization Kit (RiboBio Co.) according to the manufacturer's instructions. Skin tissue slides were deparaffinized and rehydrated, while HaCaT cells were seeded for 24 h before washing 3 times with PBS and fixation. All samples were treated with proteinase K (20 µg mL^−1^) for deproteination at 37 °C for 30 min. After the addition of pre‐hybridization buffer at 37 °C for 30 min and subsequent removal of the liquid, samples were hybridized with a custom‐designed DAP‐AS1 FISH probe (RiboBio Co.) overnight in a dark, humidified chamber at 37 °C. On the following day, the slides were washed three times with Wash Buffer I (4× SSC with 0.1% Tween‐20), followed by washes with Wash Buffer II (2× SSC) and Wash Buffer III (1× SSC) for 5 min each at 42 °C in the dark. A final wash with 1× PBS was performed at room temperature. Finally, samples were stained with PI for 10 min and treated with an anti‐fluorescence quenching agent before images were captured using a confocal laser‐scanning microscope (Carl Zeiss, Oberkochen, Germany).

### Hydroxymethylated and Methylated DNA Immunoprecipitation (hMeDIP and MeDIP) Assays

Sample DNA was extracted and ultrasonically processed to a size range of 200–1000 bp. The Hydroxymethylated DNA Immunoprecipitation (hMeDIP) Kit (Epigentek, Farmingdale, USA) and Methylamp Methylated DNA Capture Kit (Epigentek, Farmingdale, USA) were utilized according to the manufacturer's instructions. In each reaction well, the corresponding antibody (IgG, 5hmc, or 5mc) was added and incubated for 60 min at room temperature. Subsequently, the unbound material was removed, and the sheared DNA sample was added to the wells for an additional 90‐min incubation at room temperature with rotation. After being washed five times, the enriched DNA sample was eluted and collected for subsequent PCR analysis. The primers used for PCR analysis are listed in Table S2b (Supporting Information).

### Chromatin Immunoprecipitation (ChIP) Assay

HaCaT cells grown in 150 mm dishes were harvested for ChIP assays. Subsequent assays were performed using a SimpleChIP Enzymatic Chromatin IP Kit (Cell Signaling Technology, USA) according to the manufacturer's instructions. After ultrasonic treatment and centrifugation, the nucleus was obtained and incubated overnight at 4 °C with TET3 antibody (ABclonal, Beijing, CN), and normal rabbit IgG antibody (Cell Signaling Technology, USA) as the negative control. Magnetic beads were then used to capture the antibody‐protein‐DNA complexes after a 4‐h incubation at room temperature with rotation. Immunoprecipitated DNA was eluted using elution buffer and purified with the Universal DNA Purification Kit (Tiangen, Beijing, CN) as per the manufacturer's protocol. The purified DNA was subjected to quantitative PCR using SYBR Green promoter region primers for DSP. The primers used in the qPCR analysis are listed in Table S2b (Supporting Information).

### Co‐Immunoprecipitation (Co‐IP)

Cells pellets were first washed and digested with trypsin before being treated with lysis buffer containing 1 × protease inhibitor, 150 mm KCl, 20 mm Tris‐HCl (pH 7.9), 5 mm MgCl_2_, 0.2 mm EDTA, 10% glycerol, and 0.1% NP‐40. The resulting lysate was then incubated with either the S9.6 antibody or immunoglobulin G (IgG), along with protein A/G agarose beads, and subjected to gentle agitation on a rotator at 10 rpm, 4 °C for 4 h. After incubation, beads were washed three times with lysis buffer. Immunoprecipitated proteins were eluted from the beads for subsequent Western blot analysis.

### DNA:RNA Immunoprecipitation (DRIP‐qPCR)

DNA was extracted a DNA extraction kit (TIANGEN, Beijing, China). Briefly, 20 µg of DNA was fragmented with a mixture of restriction endonuclease (BsrG1, EcoR1, Hind III, SspI, XbaI) at 37 °C for 6 h, with or without RNase H1 (125 U mL^−1^). Subsequently, 4 µg of DNA was incubated overnight with 10 µg of the S9.6 antibody at 4 °C in a buffer consisting of 10 mm NaPO_4_ (pH 7.0], 140 mm NaCl, and 0.05% Triton X‐100. The resulting RNA: DNA hybrids were then captured by protein A/G agarose (Sigma, US). After thorough washing and treatment with Proteinase K (3 µg µL^−1^) at 45 °C for 60 min, the DNA was purified as previously described and subjected to qPCR analysis. The primers utilized in the qPCR are listed in Table S2c (Supporting Information).

### RNA Pull‐Down Assay

RNA in vitro transcription was conducted according to the manufacturer's protocol, where vectors containing T7 promoters were constructed, with the most downstream site being Xhol I/Xbal I. Subsequently, in vitro transcription was carried out utilizing in vitro transcription kit. The synthesized DSP‐AS1 was biotinylated using a Biotin labeling kit. For the pull‐down assays, 10 µl of beads containing 500 ng immobilized TET3 were incubated overnight at 4 °C with 500 pmol of biotin‐labeled DSP‐AS1 probes (with a specific activity of 2000 cpm ng^−1^) in a 100 µL RNA‐Protein Binding reaction system comprising 10 × Protein‐RNA binding buffer, 50% glycerol, protein lysate, and nuclease‐free water. After washing, elution, and denaturation, the released DSP‐AS1 was analyzed by SDS‐PAGE.

### Diabetic Rat Modeling

A diabetic rat model was established using streptozotocin (STZ) induction (STZ, obtained from Sigma, US) via a single intraperitoneal injection in four‐week‐old male Sprague–Dawley (SD) rats. Briefly, the rats underwent a 6‐h fast before being injected intraperitoneally with either vehicle or STZ (at a dose of 50 mg k^−1^g rat^−1^, pH 4.5). Blood glucose levels were measured 3 days post‐STZ injections, and rats exhibiting random blood glucose levels exceeding 16.7 mmol L^−1^ were considered to have successfully developed diabetes.^[^ ^40^, ^41^ ^]^ The data illustrating the successful establishment of diabetic rat models are depicted in Figure S1C (Supporting Information).

### In Vivo Wound‐Healing Assay

A full‐thickness skin wound model was established on the dorsum of male Sprague‐Dawley (SD) rats following a minimum 30‐day period of confirmed diabetes. A 2 cm biopsy punch was used to demarcate a circular wound area on the back, ≈2 cm caudal to the neck. This location minimized the influence of skin wrinkling during movements (e.g., head raising), and skin wound tissues were collected at specified time points. After wound creation using a 20 mm biopsy punch, animals were randomly assigned to treatment groups with a minimum of five rats per group. On days 0, 3, 5, 7, 9, and 11 post‐wound modeling, siNC and siDSP (4 nmol per site) were intradermally injected into the edge of the skin wound. Subsequently, the wound tissues were harvested for qRT‐PCR, IHC, H&E staining, and Masson's trichrome staining. The migration areas of wound healing were quantified using ImageJ software based on photographs taken after wound modeling. Wound areas were also quantified using ImageJ software, and wound healing rates were calculated using the formula: (initial area – final area)/initial area × 100%.^[^ ^40^ ^]^ All animal experiments were conducted in accordance with the guidelines approved by the Institutional Animal Care and Use Committee (IACUC) of Sun Yat‐sen University (approval number: 2022‐B0424).

### Patients’ Skin Tissue Specimens

Skin tissue specimens from diabetic perilesional wounds and control wounds were collected from diabetic patients and non‐diabetic patients, respectively, who underwent lower limb amputation surgery. The tissues were promptly fixed with 4% paraformaldehyde overnight at 4 °C for histological examination. Subsequently, they were embedded in paraffin and snap‐frozen in liquid nitrogen for further testing. All procedures were conducted in accordance with the principles outlined in the Helsinki Declaration II and were approved by the Ethics Committee of Sun Yat‐sen Memorial Hospital, Sun Yat‐sen University (approval number: SYSKY‐2023‐869‐01).

### Statistical Analysis

Data were presented as the mean ± SEM. Statistical analyses were performed using IBM SPSS Statistics 22.0 software. Student's t‐test, one‐way analysis of variance (ANOVA), or one‐way multivariate analysis of variance (MANOVA) followed by Fisher's least significant difference (LSD) post hoc analysis were employed to compare between groups. A significance level of ^*^ *P* < 0.05 was adopted for statistical significance.

## Conflict of interests

The authors declare no conflict of interest.

## Author contributions

C. Y., H. L., HL. L., C. S. and J. L. conceived the project with assistance from L. Y., S. C., and M. R., and designed and conducted the majority of the experiments. Y. C. collected wound skin tissues from patients, while L. W. assisted in RT‐qPCR experiments. C. Y., S. L., H. L., and HL. L. drafted the manuscript. L. Y., S. C., and M. R. supervised the study and contributed to manuscript editing. All authors reviewed and approved the final manuscript.

## Supporting information



Supporting Information

## Data Availability

The data that support the findings of this study are openly available in advs at https://doi.org/10.1002/advs.202406021, reference number advs.202406021.
