# Supplementary material for: The Diminution of R‐Loops Generated by LncRNA DSP‐AS1 Inhibits DSP Gene Transcription to Impede the Re‐Epithelialization During Diabetic Wound Healing
Source: Adv Sci (Weinh). 2025 Feb 7;12(12):2406021. doi: 10.1002/advs.202406021 (PMC11948065; doi:10.1002/advs.202406021)
Supplement: Supplementary file 1 — Supporting Information [file ADVS-12-2406021-s001.docx]

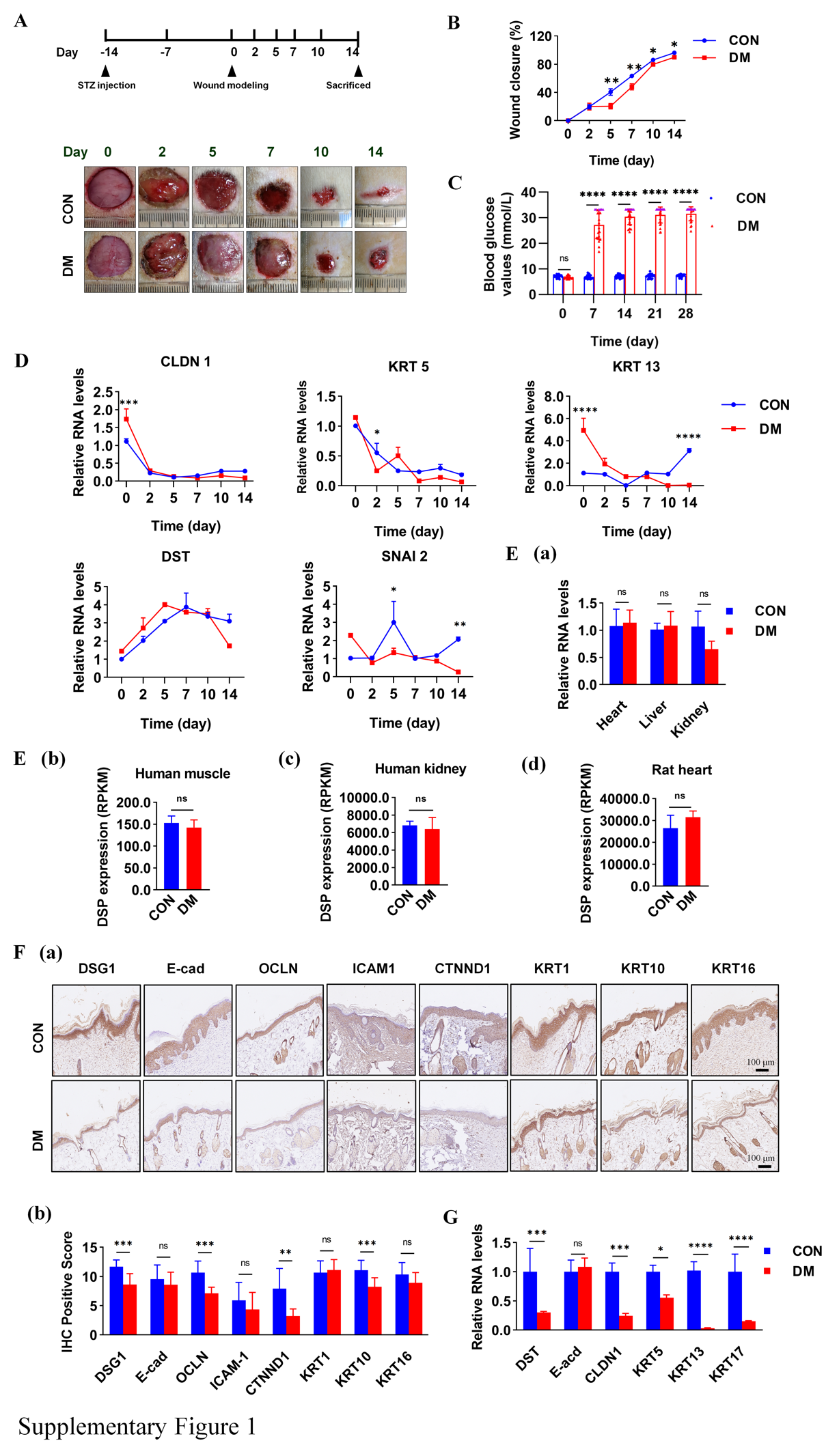


**Supplementary Figure S1. A** Representative images of skin wounds of normal and diabetic rats on day 0, 2, 5, 7, 10, and 14 of wound healing. **B** Rates of wound closure were quantified by ImageJ pro plus software and expressed as percentage of closed wound area (*n* = 4). **C** Blood glucose monitoring of diabetic rats modeling. Non-diabetic (control) (*n* = 22) and diabetic (*n* = 23) rats were tested for blood glucose concentration on day 0, 7, 14, 21 and 28 after modeling, respectively. Data were expressed as means ± SEM. P values were calculated using a two-sided t-test. Purple dots indicate blood glucose concentration greater than or equal to 33.3 mmol/L. **D** RT-qPCR analysis of claudin (CLDN 1), keratin (KRT 5), keratin (KRT 13), snail family transcriptional repressor 2 (SNAI 2), and dystonin (DST) mRNA expression levels in skin wound tissues of non-diabetic and diabetic rats on day 0, 2, 5, 7, 10, and 14 of wound healing (*n* = 4). ACTB (beta-actin) was used as an internal control. **E** **(b)** DSP expression in human muscle tissues, **(c)** human kidney tissues, **(d)** and rat heart tissues between diabetic and non-diabetic groups was explored with Gene Expression Omnibus (GEO) database and these differences were not statistically significant (*P* > 0.05). (a) DSP expression in heart, liver, kidney between diabetic rats and controls. ACTB was used as an internal control. Data were expressed as means ± SEM (*n* = 3). P values were calculated using a two-sided t-test. **F (a)** IHC staining of cell junction-related genes (DSG1, E-cad, OCLN), cell adhesion-related genes (ICAM-1, CTNND1) and differentiation-related genes (KRT1, KRT10, KRT16) protein in the epidermis (brown color). **(b)** The quantitative analysis of skin tissues of normal rats and diabetic rats by ImageJ pro plus software, respectively. **G** RT-qPCR analysis of cell junction-related genes (DST, E-cad, CLDN1), and cell differentiation-related genes (KRT5, KRT13, KRT17) of skin tissues of diabetic rats and controls, respectively. Data were expressed as means ± SEM (*n* = 4). P values were calculated using a two-sided t-test. ns (not significant, ***P*** > 0.05), **P* < 0.05, ***P* < 0.01, ****P* < 0.001, *****P* < 0.0001.

**
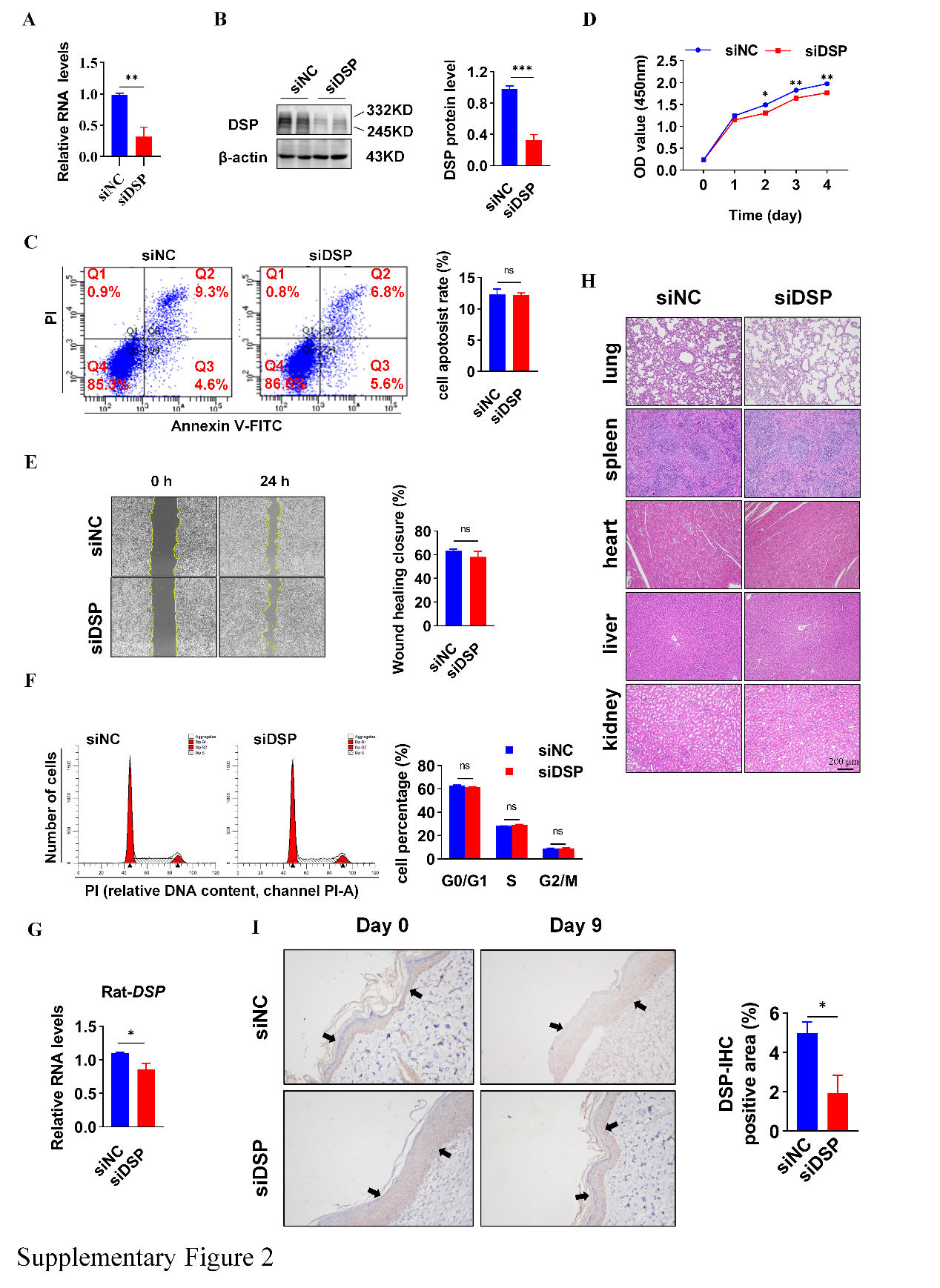
**

Fig. S2 **A&B** The RNA and protein level of DSP in HaCaT cells after treated with siNC and siDSP, respectively (*n* = 3). **C** Flow cytometry monitoring the apoptosis of HaCaT cells after treated with siNC and siDSP for 48 hours, respectively (*n* = 3). **D** CCK8 assays monitoring the proliferation of HaCaT cells after treated with siNC and siDSP, respectively (*n* = 3). **E** Representative images of wound-healing assays using HaCaT cells after treated with siNC and siDSP, respectively (Left). HaCaT cells were scratched after siRNA transfection for 24 hours. Images were captured at 0 h and 24 h after the scratch. Quantitative analysis of wound-healing assays was shown on the right (*n* = 3). **F** Flow cytometry monitoring cell cycle of HaCaT cells after treated with siNC and siDSP for 48 hours, respectively (*n* = 3). **G** The RNA level of DSP expression in the epidermis around the wounds after treated with siRNA and siDSP for 9 days, respectively, in normal rats ( *n* = 4). **H** HE staining of several organs of normal rats after treated with siRNA and siDSP for 9 days, respectively (*n* = 4). **I** IHC staining and analysis of DSP expression in skin tissues of non-diabetic rats after treated with control siRNAs (siNC) and siRNAs target to DSP (siDSP), respectively. Quantitative analysis of DSP protein (brown color) levels in the epidermis were conducted using ImageJ pro plus software (*n* = 3). P values were calculated using a two-sided t-test. ns (not significant, *P* > 0.05). **P* < 0.05, ***P* < 0.01.

**
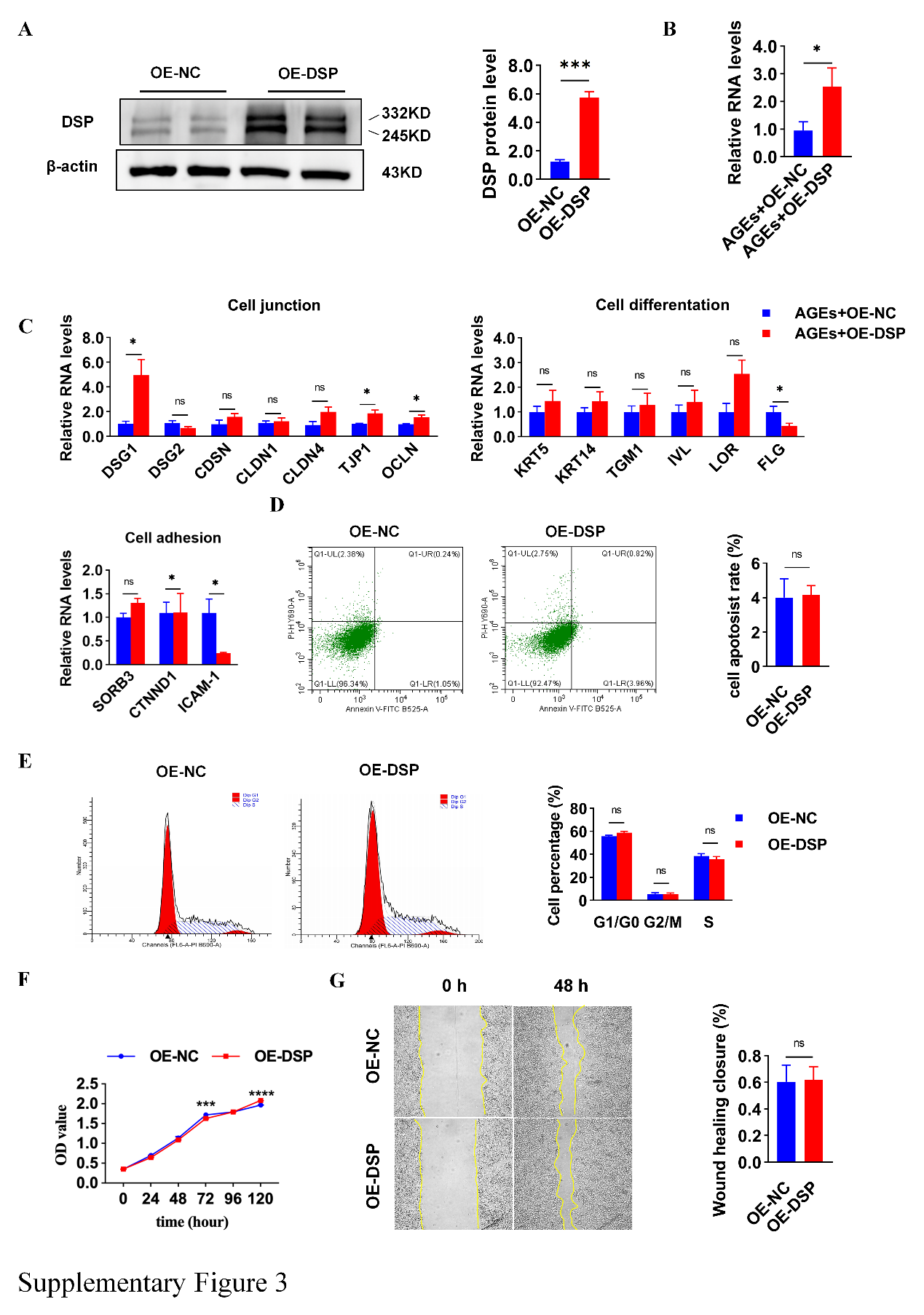
**

**Fig. S3 A&B** Establishment of stable polyclonal HaCaT cells overexpressing OE-DSP or OE-NC. Expression level of DSP protein and RNA were shown. C Effects of DSP overexpression on mRNA expression of **(a)** cell junction-related genes (DSG1, DSG2, CDSN, CLDN1, CLDN4, TJP1, OCLN), **(b)** cell adhesion-related genes (SORBS3, CTNND1, ICAM-1), and **(c)** cell differentiation-related genes (KRT5, KRT14, TGM1, IVL, FLG, LOR) in HaCaT cells (n = 3 for each group). Data were expressed as means ± SEM. **D** Flow cytometry monitoring the apoptosis of HaCaT cells after treated with OE-DSP and OE-NC, respectively (n= 3 for each group). **E** Flow cytometry monitoring cell cycle of HaCaT cells after treated with OE-DSP and OE-NC, respectively (n = 3 for each group). **F** CCK8 assays monitoring the proliferation of HaCaT cells after treated with OE-DSP and OE-NC, respectively (n = 3 for each group). **G** Representative images of wound-healing assays in HaCaT cells after treated with OE-DSP and OE-NC, respectively. HaCaT cells were scratched after transfection for 48 hours. Images were captured at 0 h and 48 h after the scratch. Quantitative analysis of wound-healing assays was shown on the right (n = 3 for each group). Data were expressed as means ± SEM. P values were calculated using a two-sided t-test. ns (not significant, *P* > 0.05). **P* < 0.05, ****P* < 0.001, *****P* < 0.0001.

**
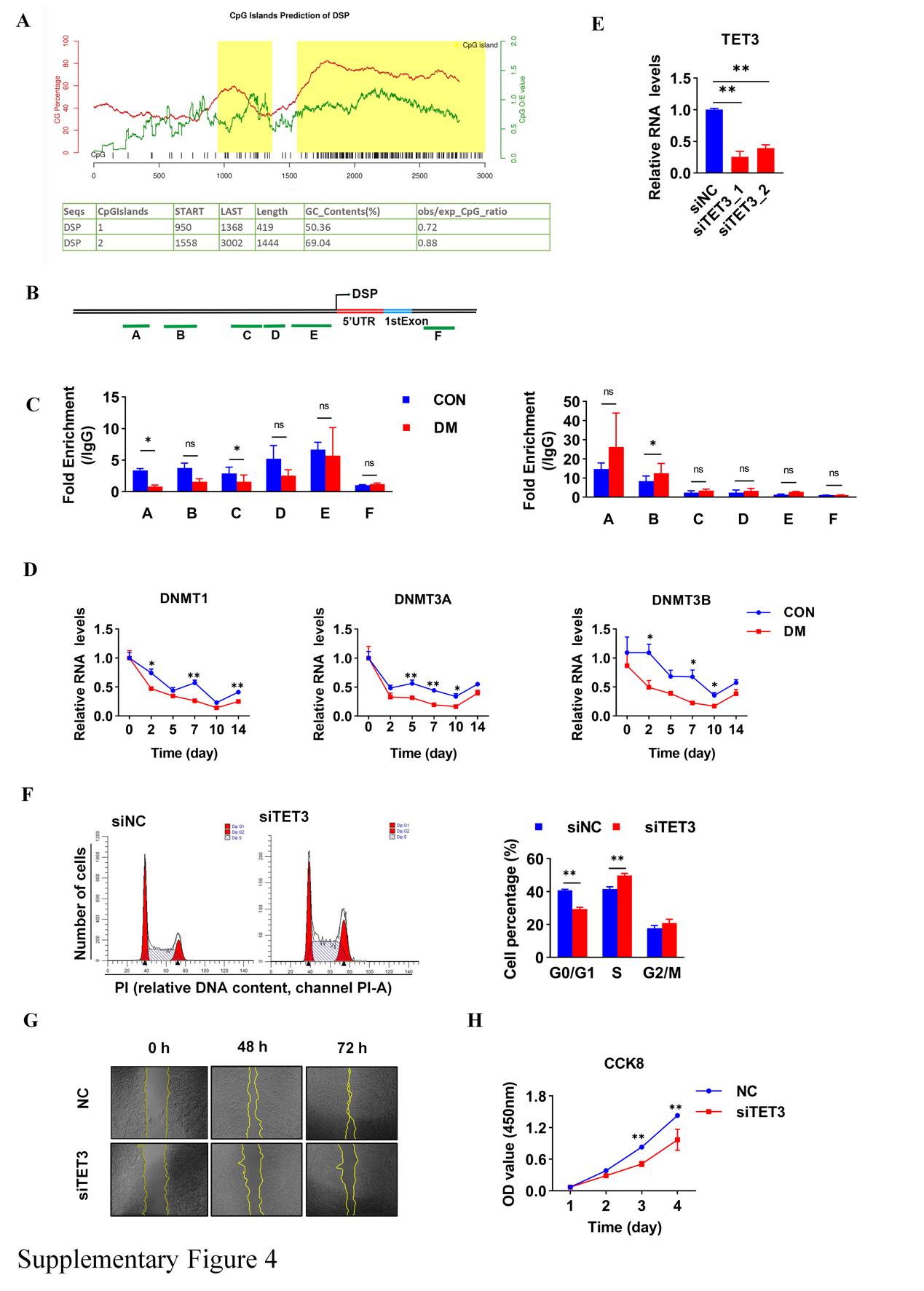
**

**Supplementary Figure S4. A** Promising result for prediction of CpG islands at the DSP promoter region using online database. Red line represents CG percentage and green line represents CpG Obs/Exp ratio value (*n* = 3). **B** Schematic representation of the six DSP amplicons targeting the DSP gene region in rats. **C** hMeDIP **(left)** and MeDIP-qPCR**(right)** analysis to assess the demethylation and methylation level at the DSP gene region in skin wound tissues of control and diabetic rats on day 14 of wound healing (*n* = 4). **D** RT-qPCR analysis of DNMT1, DNMT3A, and DNMT3B expression in skin wound tissues of normal and diabetic rats during wound healing (*n* = 4). **E** RT-qPCR assays to verify the effectiveness of siTET3 in HaCaT cells (*n* = 4). **F** Flow cytometry monitoring the cell cycle of HaCaT cells after treated with siNC and siTET3 for 48 hours, respectively. **G** Representative images of wound-healing assays using HaCaT cells after treated with siNC and siTET3, respectively. HaCaT cells were scratched after siRNA transfection for 24 hours. Images were captured at 0 h, 48 h and 72 h after the scratch. **H** CCK8 assays monitoring the proliferation of HaCaT cells after treated with siNC and siTET3, respectively. Data were expressed as means ± SEM. P values were calculated using a two-sided t-test. ns (not significant, *P* > 0.05). **P* < 0.05, ****P* < 0.001, *****P* < 0.0001.

**Supplementary Table S1. Key resources table**

| REAGENT or RESOURCE | SOURCE | IDENTIFIER |
| --- | --- | --- |
| **Antibodies** |  |  |
| Desmoplakin Polyclonal antibody | Proteintech Group | 25318-1-AP |
| S9.6 antibody | Merck | MABE 1095 |
| DSG1 Polyclonal antibody | Proteintech Group | 24587-1-AP |
| TET3 Antibody | Affinity Biosciences | DF13335 |
| TET3 Rabbit Polyclonal antibody | ABclonal Technology | A3141 |
| TET3 Polyclonal antibody | ThermoFisher | PA5-31860 |
| KRT 10 antibody | Proteintech | 16855-1-AP |
| E-cad antibody |  |  |
| KRT 16 antibody | Signalway | 55233 |
| Occludin antibody | Proteintech | 13409-1-AP |
| KRT 1 antibody | Proteintech | 16848-1-AP |
| Beta Actin Antibody | Affinity Biosciences | AF7018 |
| Normal Rabbit IgG antibody | Cell Signaling Technology | 2729 |
|  |  |  |
| **Critical Experimental Reagents** |  |  |
| SYBR® Green Premix Pro Taq HS qPCR Kit | Accurate Biotechnology | AG11718 |
| Hydroxymethylated DNA Immunoprecipitation (hMeDIP) Kit | Epigentek, Farmingdale | P-1038 |
| Methylamp™ Methylated DNA Capture Kit | Epigentek, Farmingdale | P-1015 |
| SimpleChIP® Enzymatic Chromatin IP Kit | Cell Signaling Technology | 9003 |
|  |  |  |
| **Enzymes** |  |  |
| BsrG1 | New England Biolabs | R3575V |
| EcoR1 | New England Biolabs | R0101V |
| Hind Ⅲ | New England Biolabs | R0104V |
| SspI | New England Biolabs | R3057V |
| XbaI | New England Biolabs | R0145V |
| RNase H | New England Biolabs | M0297S |
|  |  |  |
| **Experimental Models: Cell Lines** |  |  |
| Human keratinocyte cell lines (HaCaT) | Procell Life Science & Technology | CL-0090 |

| **Supplementary Table S2a. Primers for qRT-PCR** | | |
| --- | --- | --- |
| Genes | Sequence (5'-3') | |
|  | Forward | Reverse |
| **Human** |  |  |
| ATCB | CATGTACGTTGCTATCCAG | CTCCTTAATGTCACGCACGAT |
| DSP | GCAGGATGTACTATTCTC | CCTGGATGGTGTTCTGGTTCT |
| TET3 | CCACAAGGACCAGCATA | CTCGCTACCAAACTCATCCGT |
| DSG1 | CCTGCTGCTTGCTTT | GGTTATTGGGCTCGTC |
| DSG2 | TGCTGCTTCTCCTGATCTGC | GCTGCTGTGTTCCTCTCTGT |
| CDSN | CGTATCACCTCCCCTAACG | GAGGAGTAGCTGACCTGGGA |
| CLDN1 | CCTCCTGGGAGTGATAGCAA | GGCAACTAAAATAGCCAGAC |
| CLDN4 | GGGGCAAGTGTACCAACTG | GACACCGGCACTATCACCA |
| OCLN | GCATCGCTGTATTCGC | TCCATAGGCTCTGTCCC |
| TJP1 | CAACATACAGTGACGCTTCA | CACTATTGACGTTTCCCCACTC |
| KRT5 | TCAACAAGCGTACCACTGCT | CTGCTACCTCCGGCAAGAC |
| KRT14 | GGGTTTGCCTCCTTCTCC | TGGGCAGATGAAAGGT |
| FLG | TGAGGGCACTGAAAGGCA | TGGCCACATAAACCTGGGTC |
| TGM1 | ATGATGTGTCACGCCACCTT | GCATGCAATAGGGAAAGCCG |
| IVL | GTTCCTCCTCCAGTCAATAC | TGCTCACATTCTTGCTCAG |
| LOR | AACAGTATCAGTGCCAGAG | GGTCTGCTGAGAGGAGTA |
| ICAM-1 | CGACTGGACGAGAGGGATT | GATAGGTTCAGGGAGGCGT |
| SORBS3 | CCGCGAACTTTTCCGGAGG | CCGCGAACTTTTCCGGAGG |
| CTNND1 | TGAGTGGTTCTCCAGAGGG | AGGCTTCTAGGATGGCAGGA |
| **Rat** |  |  |
| ACTB | GTCCACCCGCGAGTACAAC | GGATGCCTCTCTTGCTCTGG |
| DSP | ATGTTGAAGGTCTACGAAG | GTCCCACAATCTAAAGTCTA |
| TET1 | ACTGAAGAATGACGCAACA | AGGAGAGGTGAGGAAGGA |
| TET2 | CAGCACACCCTCTCAAGAT | TGGTTTTCTGCACCGCAATG |
| TET3 | CAGCCGCATCTCTGGAGG | TTCTTTCGCCCATCTCCACC |
| PARD3 | AGCCAAGCCAGATGCAGA | ACTGTAGGGGACAGCTGGTA |
| FN1 | GGTCCGGGACTCAATCCAA | GACAGAGTTGCCCACGGTA |
| E-cad | CGAGAGCTACACGTTCA | GGGTGTCGAGGGAAAAATAG |
| N-cad | TCAGGCGTCTGTAGAGGCTT | ATGCACATCCTTCGATAAG |
| VIM | AGTCCACTGAGTACCGGAG | CATTTCACGCATCTGGCGTTC |
| DNMT1 | AGGAGGGCTACCTGGCTAA | CGTCTCCATCTTCGTCCTCG |
| DNMT3A | CGGCCATACGGTGGAGCC | CAGACCTTTAGCCACGACCC |
| DNMT3B | CCGCTTCCTCGCAGCAG | TGGGCTTTCTGAACGAGTC |
| DSG1 | TACAGCAGTTCAGAGTCATT | ACATTGGTGGAGGTCATAC |
| OCLN | GTATAAGTCACCGCCTCTG | ACTCTTCGCTCTCCTCTC |
| KRT1 | CTTCTTCTCCACACTCTACC | TGCTTCTTGACGCTATCG |
| KRT10 | GCTTCGGTGGAGGATATG | CATTGGCATTGTCAGTTGT |
| KRT16 | CCTATTCTTCCCGCGAGGT | GGGAGATAGCTGGGAACTGC |

| **Supplementary Table S2b. Primers for ChIP-qPCR and hMeDIP-qPCR** | | |
| --- | --- | --- |
| Genes | Sequence (5'-3') | |
|  | Forward | Reverse |
| **Human** |  |  |
| 1 (DSP locus,  -1247 ~ -1061) | AATGGACCTGGCGTTGAA | AACCACGACTTCCTGGAA |
| 2 (DSP locus,  -1561 ~ -1463) | CACCCTGGGAAGAAACC | AGGAGCGGGAGGAAA |
| **Rat** |  |  |
| A (DSP locus,  -1315 ~ -1502) | ATCCGTACCCACAAGTCAGC | TTTGTCTTTTGCACGGTCTG |
| B (DSP locus,  -981 ~ -1211) | GCTGGTTAAAGCAACCCAAA | AGTGGGCGAGCTAACAAGTG |
| C (DSP locus,  -514 ~ -742) | TCCTGTAATCCTGGCACTCC | GAATGTTCTGGCTGGGTGTT |
| D (DSP locus,  -400 ~ -533) | TTCCATCGGATGACAGACAA | GGAGTGCCAGGATTACAGGA |

| **Supplementary Table S2c. Primers for DRIP** | | |
| --- | --- | --- |
| Genes | Sequence (5'-3') | |
|  | Forward | Reverse |
| **Human** |  |  |
| DSP | CACCCCACCCTGGGAAGA | CAGGAGCGGGAGGAAAGC |

| **Supplementary Table S3. Effects of siNC and siDSP on plasma biochemical indexes of the rats**^1^ | | | | |
| --- | --- | --- | --- | --- |
| Items | siNC | siDSP | SEM | *p* Value |
| ALT, U/L | 44.94 | 49.82 | 19.75 | 0.77 |
| AST, U/L | 115.76 | 137.78 | 34.67 | 0.44 |
| TG, mmol/L | 0.61 | 0.82 | 0.35 | 0.49 |
| TBIL, μmol/L | 11.49 | 13.54 | 2.80 | 0.37 |
| BUN, mmol/L | 6.56 | 8.75 | 3.50 | 0.45 |
| CR, μmol/L | 27.99 | 30.23 | 7.19 | 0.71 |
| CK, U/L | 564.88 | 532.15 | 270.82 | 0.89 |
| CK-MB, U/L | 72.28 | 51.6 | 22.93 | 0.26 |

^1^Values are means of 8 samples with 4 rats per replicate. siNC: the control group treated with NC siRNAs; siDSP: the group treated with siRNAs targeting DSP; ALT: alanine aminotransferase; AST: aspartate aminotransferase; TG: triglyceride; TBIL: total bilirubin; BUN: blood urea nitrogen; CR: creatinine; CK: creatine kinase; CK-MB: creatine kinase isoenzyme; SEM: standard error.

| **Supplementary Table S4. siRNA target sequences** | |
| --- | --- |
| siRNA | Target sequence |
| siDSP-Homo | GCTAGTAGATTCTGGTATA |
| siDSP-Rat | CTGGATCAAAGCAGACATGC |
| siDSP-AS1 | CTGAGTGTTAACTGCAAGA |
| ASODSP-AS1 | CTCTCCGGAACTCAGAGCTG |
| si-h-TET3_101 | GCAACTCCTAGAACTGAGT |
| si-h-TET3_102 | CGATTGCGTCGAACAAATA |

**Supplementary Table S5. Table The position and primers sequence of 6 amplicons in DSP promoter region of rats**

| Amplicon | Primer sequence | Distance from transcription start site |
| --- | --- | --- |
| A | F: ATCCGTACCCACAAGTCAGC | -1315 ~ -1502 |
|  | R: TTTGTCTTTTGCACGGTCTG |  |
| B | F: GCTGGTTAAAGCAACCCAAA | -981 ~ -1211 |
|  | R: AGTGGGCGAGCTAACAAGTG |  |
| C | F: TCCTGTAATCCTGGCACTCC | -514 ~ -742 |
|  | R: GAATGTTCTGGCTGGGTGTT |  |
| D | F: TTCCATCGGATGACAGACAA | -400 ~ -533 |
|  | R: GGAGTGCCAGGATTACAGGA |  |
| E | F: AGCCTGAGCAGGGACGAG | -76 ~ -360 |
|  | R: GGAAAAGCCCTTTACAGGATTC |  |
| F | F: ATCCACTGCTCCCTTCCAC | +812 ~ +617 |
|  | R: GGTCTAGGCCGAGGAAAGTG |  |
